# Current biomarkers of canine mammary tumors

**DOI:** 10.1186/s13028-018-0417-1

**Published:** 2018-10-29

**Authors:** Ilona Kaszak, Anna Ruszczak, Szymon Kanafa, Kamil Kacprzak, Magdalena Król, Piotr Jurka

**Affiliations:** 1 Department of Small Animal Diseases with Clinic, Veterinary Medicine Faculty, Warsaw University of Life Sciences, Nowoursynowska 166, 02-787 Warsaw, Poland; 2 Department of Physiological Sciences, Veterinary Medicine Faculty, Warsaw University of Life Sciences, Nowoursynowska 166, 02-787 Warsaw, Poland

**Keywords:** Biomarkers, Bitch, Canine mammary gland, Canine mammary tumors, Dog, Human breast cancer, Neoplasia, Veterinary oncology

## Abstract

Mammary tumors are the second most common neoplasia in dogs. Due to the high similarity of canine mammary tumors (CMT) to human breast cancers (HBC), human biomarkers of HBC are also detectable in cases of CMT. The evaluation of biomarkers enables clinical diagnoses, treatment options and prognosis for bitches suffering from this disease. The aim of this article is to give a short summary of the biomarkers of CMT based on current literature. Very promising biomarkers are miRNAs, cancer stem cells, and circulating tumor cells, as well as mutations of the breast cancer 1 gene (BRCA1) and breast cancer 2 gene (BRCA2). Until now, the most studied and reliable biomarkers of CMT have remained antigen Ki-67 (Ki-67), endothelial growth factor receptor, human epidermal growth factor receptor 2 (HER-2), estrogen receptor, progesterone receptor and cyclooxygenase 1 (COX-2), which can be detected in both serum and tissue samples using different molecular methods. However, carcinoembryonic antigen and cancer antigen 15-3 (CA 15-3), while poorly studied, seem to be good biomarkers, especially for the early detection and prognosis of CMT. We will also mention the following: proliferative cell nuclear antigen, tumor protein p53 (p53), E-cadherin, vascular endothelial growth factor, microRNAs, cancer stem cells and circulating tumor cells, which can also be useful biomarkers. Although many studies have been conducted so far, the estimation of biomarkers in cases of CMT is still not a common practice, and more detailed research should be done.

## Background

With the increase in life expectancy in both humans and dogs, the incidence of cancers has increased as well [1]. Mammary neoplasia is the most frequently diagnosed tumor in bitches; therefore, they represent a significant clinical problem [2–5]. Among canine mammary tumors, (CMT) approximately 50% are malignant [2, 3, 5]. The most common tumor type among them is tubular carcinoma (adenocarcinoma), followed by papillary carcinoma, solid carcinoma, complex carcinoma and carcinosarcoma (Table 1) [3, 4]. The benign mammary tumors are mostly fibroadenomas, ductal papillomas, benign mixed tumors and simple adenomas [3, 4]. It is also common to find more than one tumor type in different mammary glands of the same patient [2]. In contrast breast cancers in women are usually malignant, and the most common tumor type is invasive ductal carcinoma, followed by carcinoma in situ [6]. The grade of malignancy is based not only on tumor type but also on the presence of significant nuclear and cellular pleomorphism, mitotic index, the presence of necrotic areas, peritumoral and lymphatic invasion and regional lymph node metastasis [7]. CMT occur in elderly bitches, usually between 8 and 10 years old [2–5]. This problem is especially significant in Europe, where bitches are usually spayed at an older age [2]. The etiology of CMT is still unknown. However, some risk factors have been indicated, e.g., hormonal, nutritional and genetic factors [2, 8–10]. It has been demonstrated in several studies that certain breeds have a genetic tendency to suffer from CMT, e.g., Miniature Poodles, Dachshunds, Malteses, Yorkshire Terriers, Cocker Spaniels and German Shepherds [2, 3, 5]. However, these predispositions may vary depending on the geographical location. However, no common mutations in dogs with CMT have been detected so far [2]. One of the main risk factors for tumor growth is sex hormones [8–10]. The risk of mammary tumors increases depending on the time of spaying. If the bitch is spayed before her first ovarian cycle, the risk is 0.5%, but when she is spayed just after or at any time later, the risk is 8% and 26%, respectively [2, 3, 5, 8]. Apart from that, false pregnancies and the use of progestins as contraception can contribute to tumor formation [2, 5]. Usually, the owners of animals notice tumors when macroscopic changes in the mammary gland are already visible [2]. The prognostic evaluation is usually based on the clinical stage, tumor size, lymph node status, radiographic evidence of distant metastases, lymphatic or vascular invasion and histopathological examination of the tumor according to the WHO guidelines after tumor removal [2, 7, 11–14]. So far, the only effective treatment is surgery, which consists of the removal of altered glands and local lymph nodes. Usually, at the same time, an ovariohysterectomy (OHE) is also performed. However, recent studies have demonstrated that not all CMT cases benefit from OHE [15]. The standard patient follow-up protocol includes clinical examination and a basic panel of blood tests, together with an X-ray check every 3 months. In most cases of malignant CMT, chemotherapy or radiotherapy is performed. Nevertheless, both chemotherapy and radiotherapy are costly, and there is limited information about their efficacy in the treatment of CMT [16]. Because of this, the early detection of CMT seems to be crucial for the patients’ outcome. Unfortunately, little is known about cancer biomarkers. Determination of biomarkers in dogs could be a milestone for the early diagnosis of neoplasia and for evaluation of the progression of disease and its response to chemotherapy.

**Table 1:** Mammary gland neoplasias in bitches [7]

Malignant	Benign
1. Simple carcinoma* Tubopapillary carcinoma Tubular carcinoma adenocarcinoma* Solid carcinoma Anaplastic carcinoma 2. Carcinoma in situ 3. Complex carcinoma* 4. Special type of carcinomas Spindle cell carcinoma Squamous cell carcinoma Mucinous carcinoma Lipid-rich carcinoma Inflammatory carcinoma 5. Carcinoma arising in a mixed tumor 6. Carcinosarcoma 7. Sarcoma Fibrosarcoma Osteosarcoma Other sarcomas 8. Comedocarcinoma	1. Adenoma* Simple adenoma Intraductal papillary adenoma Complex adenoma Basaloid adenoma 2. Fibroadenoma* 3. Benign mixed tumor* 4. Ductal papilloma 5. Myopithelioma 6. Mammary hyperplasia and dysplasia Ductal hyperplasia Lobular hyperplasia Cysts Duct ectasia Focal fibrosis Gynecomastia

* The most frequent types

Biomarkers are usually proteins that can be measured in blood or other tissues (e.g., in tumor tissue) and can give information about the presence of a disease, results of treatment or further prognosis for the patient [17, 18]. Every cancer cell expresses specific proteins that are called tumor-associated antigens. In patients with tumors, these antigens are called biomarkers if they can be detected in other tissues, serum or urine in concentrations that are different from normal [17, 18]. Therefore, we can distinguish between serum and tissue/cellular biomarkers. In human medicine, biomarkers play a role in breast cancer diagnosis and follow-up. Human breast cancer (HBC) has similar immunohistochemical features to canine mammary tumors and expresses the same substances; therefore, the determination of the same markers may be considered [19]. Nevertheless, canine mammary cancers have a very heterogenous morphology and biology, and the choice of the most appropriate biomarker remains the biggest challenge.

In this article, we briefly describe the most reliable biomarkers of canine mammary tumors [17, 19–22] that are the most frequently determined in mammary gland tissue by immunohistochemical methods. However, we also mention possibilities to determine additional serum biomarkers by other molecular techniques.

## Search strategy

This article is a literature review and is based on an examination of current veterinary and medical literature. A literature search was performed in PubMed (http://www.ncbinlm.nih.gov/pubmed) using the terms “canine mammary tumor biomarkers”, “human breast cancer biomarkers”, “Ki-67”, “PCNA”, “p53”, “E-cadherin”, “CEA”, “CA 15-3”, “VEGF” EGFR”, “HER-2”, “ER”, “PR”, “COX-2”, “BRCA 1”, “BRCA 2”, “miRNA”, “CSCs” and “CSCs”. Only papers written in English and published within the last 20 years, until November 2017, were included. The articles most relevant to our review were selected.

## Biomarkers of cancer cell proliferation and apoptosis

An estimation of a tumor’s proliferation potential is useful for determining tumor malignancy. A high proliferation rate is related to fast tumor growth and its ability to cause local and distant metastases. Ki-67 and PCNA are biomarkers of proliferation, whereas p53 is a marker of neoplastic transformation and apoptosis.

### Ki-67

Ki- 67 is the most studied biomarker of canine tumor proliferation and apoptosis and is detected in many tumor types. In mammary neoplasia, it is the most frequently used prognostic biomarker. Ki-67 is a nuclear, non-histone protein, which can only be detected in the cell nucleus during interphase, and during mitosis. Ki-67 is relocated to the surface of chromosomes. It can already be detected in the G1 phase of the cell cycle and increases in the S and G2 phases. It has the highest expression in the M phase, and its expression disappears rapidly after mitosis [23–28]. In G0 phase it is undetectable. It is a serum as well as a cellular biomarker, and different molecular techniques can be used for measuring its expression [23–30]. Determination of mitotic index (MI) in the hematoxylin and eosin stain routinely used in histology is not as precise as immunohistochemistry (IHC) Ki-67 staining, as MI shows only the number of cells undergoing mitosis, and Ki-67 in IHC staining is expressed in different phases of the cell cycle [26]. It can even be evaluated in cytological samples from a fine needle biopsy of the canine mammary gland, which creates an option for early tumor diagnosis [31].

In human medicine, high expression of Ki-67 in tumor tissues is correlated with a poor prognosis, but at the same time, it has been demonstrated that patients with a high Ki-67 responded well to chemotherapy, probably due to the high proliferative activity [23]. Although it seems to be a good prognostic marker, the American Society of Clinical Oncology does not recommend its use in clinical practice due to the lack of uniformity in laboratory techniques and analysis [32]. Immunohistochemical evaluation of Ki-67 expression, together with the assessment of estrogen receptor (ER), progesterone receptor (PR) and human epidermal growth factor receptor 2 (HER-2) levels, is used to define different subtypes of breast cancer [32].

So far, only one study has been conducted to evaluate its value as a serum biomarker, using a canine-specific ELISA kit for its determination [33]. The Ki-67 was undetectable in healthy dog serum, while serum levels in tumor bearing dogs correlated positively with tumor grade [30]. The levels of Ki-67 in IHC staining (only cells with nuclear staining were recorded as positive) were determined and are significantly lower in benign than in malignant mammary tumors, and high values correlated positively with metastasis, poor prognosis and poor survival [24, 25, 28]. Ki-67 expression is the highest in tumors with poor clinical and histopathological characteristics, such as large tumor size, inflammation and ulceration of the mammary gland, invasion into the surrounding tissues, and metastases to the lymph nodes [25, 34, 35]. Another study demonstrated that the expression level of Ki-67 measured in metastases to lymph nodes had a positive correlation with its expression in the tumor tissue [36]. However, levels of Ki-67 in older bitches tend to be lower than in younger bitches, probably due to the fact that tumor proliferation processes are slower at older ages [25].

This biomarker seems to be very promising; however, more studies on a more homogenous group should be carried out.

### PCNA

PCNA is also a marker of the proliferation index (PI). PCNA is an auxiliary protein of DNA polymerase δ, which is expressed in the nuclei of cells during the DNA synthesis phase of the cell division cycle. It is involved in the DNA repair process, cell cycle control, chromatin assembly and in RNA transcription [19, 32]. It increases in the late G1 phase, reaches its peak in the S-phase and remains high in the G2 and M- phases due to its long half-life [26, 37]. It is responsible for maintaining the cell cycle continuity. PCNA has a role on both the leading and lagging strands [32].

In human medicine, PCNA is considered a good proliferation biomarker, but only when evaluated together with other HBC biomarkers, such as ER, PR, Ki-67 or HER-2. The disadvantage of PCNA as a marker is that it is not proliferation specific, but it is also involved in DNA repair [32, 37].

In veterinary medicine, the PCNA level is frequently evaluated in cases of mammary cancer [20]. PCNA expression in IHC staining (cells with nuclear staining were recorded as positive) also has a positive correlation with tumor size, tumor histological type, differentiation grade, nuclear grade, mitotic index, histological grade of malignancy, and lymph node metastasis [29]. Additionally, similar to Ki 67, its expression is greater in tumors that show more malignancy, have a large tumor size, skin ulceration, histological grade II or III, and presence of regional lymph node metastases [29]. In one study, analysis of the expression of PCNA was also confirmed in non-neoplastic, adjacent tissues, suggesting the presence of early micrometastases and higher tumor aggressiveness, which was histologically confirmed [29]. Therefore, the high expression of PCNA is associated with poor disease prognosis and decreased survival [29].

However, it is believed that PCNA expression may be stimulated by cytokines (e.g., transforming growth factor, epidermal growth factor, platelet-derived growth factor) without inducing DNA synthesis and may also be due to PCNA’s association with DNA repair. Therefore, its expression may not be specific for cancer. Because of this, it is also recommended to evaluate the expression of PCNA in combination with other biomarkers, especially Ki-67, as its expression is restricted to the cell cycle [26].

### Protein p53

P53 is an important biomarker of cell neoplastic transformation, cell division and apoptosis programs [19, 38–43]. This protein is responsible for the control of the cell cycle and apoptosis, and it is a suppressor of tumor development. During the neoplastic process, it accumulates in tumor tissue, and due to its gene mutation, it starts to play a role as an oncogene. Mutations of the p53 gene are thought to be the most common genetic alteration in HBC, as well as CMT [2]. Its mutation is associated with tumor progression [42]. Mutated p53 also stimulates the expression of p21, a protein that belongs to cyclin-dependent kinases. However, other factors can also induce p21 expression independent of p53 [41] Still, the relationship between the expressions of those two important proteins is unclear.

In human patients, a mutation of p53 is found in the majority of HBC, especially in more aggressive types, such as triple negative breast cancer [44, 45]. High expression of p53 correlates positively with a worse prognosis and shorter survival time [45].

In veterinary medicine, high expression of p53 is also generally associated with poor overall survival. Recent studies showed a higher p53 expression in large-breed dogs, suggesting that there may be heritable gene mutations and some breed predispositions, but further studies in this field must be conducted [41].

Both in human and veterinary patients, p53 overexpression (mainly evaluated by IHC in tumor tissues) correlates with an unfavorable prognosis. Although several mutations of p53 have been identified, its functionality is still unclear and there is no evident association between these mutations and incidence of malignant CMT [21].

## Biomarkers of metastatic potential of the tumor

Another important group of biomarkers are those that indicate the metastatic potential of the tumor. The ability of the tumor to metastasize depends on the cells adhesions to each other or adjacent tissues. The strength of those connections is measured by the levels of expression of the proteins involved in these processes. There are various types of adhesion molecules, such as integrins, selectins, immunoglobulin-like particles and cadherins. Cadherins are calcium-dependent transmembrane proteins and are good indicators of tumor metastasis. Additionally, CEA and CA 15-3 are glycoproteins involved in intracellular adhesions and represent one of the most often evaluated biomarkers of HBC.

### E-cadherin

Cadherins are calcium-dependent transmembrane glycoproteins and are responsible for cell–cell adhesions [46]. Their function is to maintain the normal structure of a tissue. Among cadherins, there are E-, P- and N-cadherin. P-cadherin (placental-cadherin) is expressed in myoepithelial cells, and its overexpression in high-grade tumors suggests tumor aggressiveness and a poor prognosis. However, the most frequently evaluated cadherin is E-cadherin (epithelial-cadherin), which is involved in epithelial cellular adhesion [46].

In human patients, the downregulated expression of E-cadherin is correlated with tumor histological grade, tumor size, and lymph node status, and overall, it is a predictor of a worse prognosis [47]. The loss of E-cadherin expression is a sign of epithelial-mesenchymal transition (EMT), which induces cell dissemination through an increase in cell migration and invasion [48].

In veterinary medicine, because most canine mammary tumors are of epithelial origin, a lowered expression of E-cadherin is related to increased tumor development and disease progression, tumor malignancy, aggressiveness of metastases and short overall survival [19, 30, 46]. E-cadherin binds to a group of interconnected proteins called catenins, often with β-catenin. β-catenin interacts with the cytoplasmic domain of E-cadherin and enables clustering into junctional structures. Any dysfunction of that complex reduces cell adhesion. β-catenin is a cytoplasmic protein that is degraded in the neoplastic process, which results in its accumulation in the cytoplasm and nucleus of tumor cells. Its expression is associated with tumor progression, metastases and a worse prognosis for the patient [20]. Normally, levels of β-catenin expression in blood are determined together with E-cadherin.

In both human and animal patients, low expression of E-cadherin (evaluated by IHC in tumor tissues) is correlated with a worse prognosis; however, it should be evaluated together with other biomarkers, such as Ki67. Nevertheless, it is not the most commonly evaluated biomarker of canine mammary tumors.

### CEA

CEA is a glycoprotein that is involved in intracellular adhesion. It is a glycoprotein produced by gastrointestinal mucosa, localized in epithelial cell membranes in small amounts, and it is overexpressed by the cancer cells of the colon, breast and lungs.

In humans, CEA was one of the first identified tumor biomarker of breast cancer [49]. Currently, together with CA 15-3, it is the most frequently used biomarker in HBC [49]. It is usually measured in serum using various immunochemical methods, such as radioimmunological methods (RIA) or electrochemistry luminescence immunoassay (ECL) [49, 52]. There is a positive correlation between changes in CEA blood levels and response to treatment in patients with metastases of breast cancer. It is also a useful marker for early detection of recurrence and metastasis [50–53]. CEA overexpression corresponds positively with tumor clinicopathological features, such as tumor size, tumor grade and lymph node status [52]. However, several studies have confirmed that only results of the measurements of levels of both glycoproteins (CEA and CA 15-3) may be considered sensitive and specific.

In veterinary medicine, there have only been a few studies carried out that have confirmed that CEA is detectable in canine tissues as well [49, 54]. In dogs with mammary gland tumors, serum levels of CEA were elevated compared to healthy dogs [54]. In reference to human CEA serum values, in veterinary medicine, these values are approximately ten times smaller and ranged from 0.00 to 0.23 ng/ml. CEA can be measured in both serum and tissue samples using different molecular techniques. Importantly, human kits can be used to evaluate serum levels of CEA and CA 15-3 [54].

Although it is a commonly used biomarker in human medicine, its usefulness in the early detection of primary breast cancers remains doubtful due to its low sensitivity and specificity. Therefore, only CEA determination, together with Cancer Antigen 15-3 (CA 15-3), may be useful in an early cancer diagnosis in both humans and dogs [52–56]. However, CEA plays an important role in the follow-up. Several studies have agreed that elevated levels of CEA, determined alone or together with CA 15-3, as well as with other biomarkers such as HER 2, may be a factor in recurrences and metastases [51–53, 55]. To confirm its application in veterinary medicine, further studies must be done.

### CA 15-3

CA 15-3 (also called Mucin 1 or MUC 1) is a large transmembrane glycoprotein, a product of the *mucin 1* gene, expressed in the apical plasma membrane [56]. However, during malignant transformation, it can be overexpressed on the membrane surface, as well as in the cytoplasm [57]. In humans, it is frequently overexpressed in many adenocarcinomas [56]. This antigen possesses two epitopes that are recognized by two monoclonal antibodies: glycoprotein DF3 *(mucin* 1 derived) and 115d8 [56]. During the malignancy process, MUC1 often acts as an anti-adhesive molecule and enables the detachment of malignant cells; therefore, it increases the metastatic and invasive potential of tumor cells [56].

The CA 15-3 is a very good biomarker for monitoring therapy outcomes and detecting recurrences and metastases in humans. It correlates positively with clinical and pathological tumor features, such as lymph node status, tumor size and disease stage [51]. However, as stated before, it is recommended to determinate CA 15-3 together with CEA. [51–53].

In veterinary medicine, it has been proven that CA 15-3 has a positive correlation with tumor grade (higher CA 15-3 serum concentrations were found in grade II and III carcinomas than in grade I carcinomas) [56]. Additionally, high levels of CA 15-3 were correlated with a poor clinical stage and bad prognosis. Apart from that, studies confirmed that tumor size, skin ulceration, necrosis, inflammation and histological type of mammary cancer have no relation to the serum levels of CA 15–3. CA 15-3 was also expressed in normal mammary tissues, as well as in benign tumor tissues [56].

Although CA 15-3 is the most widely used serum biomarker in patients with breast cancer, due to its low sensitivity, determination of CA 15-3 in the diagnosis of primary breast cancer is precluded. Therefore, it is used in follow-up monitoring, especially when other biomarkers, such as CEA, are evaluated at the same time. In veterinary medicine, published studies were made based on a small number of heterogeneous patients. Therefore, more studies need to be done before it can be considered an adequate biomarker.

## Biomarkers of angiogenesis

Every neoplastic process includes the formation of new blood vessels on the base of already existing vessels. This is essential for delivering nutritional supplies for the tumor and maintaining tissues homeostasis [58]. With tumor growth, the angiogenic process is advanced. Therefore, such biomarkers may be crucial for the determination of tumor progress or the presence of metastases. It has been shown that malignant CMT have significantly more blood vessel formation than benign tumors [59]. Common biomarkers of angiogenesis are vascular endothelial growth factor (VEGF), epidermal growth factor receptor (EGFR) and von Willebrand factor VIII or CD 31 [20]. VEGF is a protein, and its expression in tumors is responsible for angiogenesis and lymphangiogenesis, whereas EGFR is a tyrosine kinase receptor and acts as a promotor of cell migration and invasion [20, 60]. Von Willebrand factor VIII is a glycoprotein that participates in platelet adhesion and is expressed in both healthy and neoplastically transformed blood vessel endothelial cells and lymphatic vessels [20]. CD31, also called platelet/endothelial cell adhesion molecule, is a protein that plays a role in inflammatory processes and is expressed in vascular endothelium of arteries, veins and capillary blood vessels [20].

### VEGF

VEGF is the most frequently used biomarker in human medicine, as it controls angiogenesis. VEGFs are proteins encoded by four genes: VEGF-A, VEGF-B, VEGF-C and VEGF-D. VEGF-A and VEGF- B are responsible for angiogenesis, whereas VEGF-C and VEGF-D are responsible for lymphangiogenesis [60]. In healthy tissues, VEGF is responsible for the formation of new vessels and regulates their functions and structure. VEGF stimulates cell migration and endothelial proliferation and increases microvascular permeability, which allows cells to escape from the blood vessels and form distant metastases; it also inhibits the regression of newly formed vessels stimulates endothelial cell invasion to form new vessels and, at the same time, inhibits apoptosis [58, 60–63].

In human medicine, increased expression of VEGF (evaluated by IHC in tumor tissues as well as by ELISA in serum samples) is found in different tumors, including: lung, gastric, ovarian, endometrial, and breast. Its overexpression is related to tumor growth and metastasis, while its down-regulation results in suppression of tumor development [64, 65].

In CMT, it has been shown that increased VEGF serum levels correlate positively with a worse clinical stage, bad prognosis and lower survival rate. It is usually increased in cases of more malignant tumors with infiltrative growth. This is why it is considered a marker of tumor development and metastasis. VEGF is also increased in tumors with histologically diagnosed necrosis [61].

In both human and veterinary medicine, VEGF seems to be both a good serum and cellular biomarker. It is especially useful for the early detection of HBC [64, 65], as well as CMT. The same studies also claimed that VEGF sensitivity is increased when combined with CA 15-3 determination.

### EGFR (HER 1)

EGFR is a transmembrane tyrosine kinase receptor and belongs to the family of human epidermal growth factor receptors (HER). The family consists of EGFR (HER-1/ErbB-1), HER-2/ErbB-2, HER-3/ErbB-3, and HER-4/ErbB-4, which are usually located on cell membranes. They are transmembrane receptors that contain three domains: an extracellular ligand-binding domain, a transmembrane domain, and an intracellular domain with tyrosine kinase activity [66]. EGFR in malignant tumors is thought to be a promoter of cancer cell migration and invasion [67].

The EGFR increases angiogenesis and metastasis and is associated with poor clinical outcomes in breast cancer patients [68].

Several studies have revealed a role of EGFR in malignant tumor development and progression of the disease in canine mammary tumors [68, 69]. High expression of EGFR (evaluated by IHC in tumor tissues) is associated with a large tumor size, tumor necrosis, high mitotic index, the histological grade of tumor malignancy and a poor clinical stage [68–70]. An association between EGFR expression and the dog’s age was also found [69]. There is a positive correlation between EGFR and CD31 and between EGFR and COX-2 expression [68, 70]. The EGFR can regulate the synthesis and secretion of other angiogenic factors, such as VEGF. There are also studies on the use of specific EGFR tyrosine kinase inhibitors in cancer treatment for both humans and dogs [68]. EGFR inhibitors alone or together with COX-2 inhibitors seem to be a good treatment option for advanced malignant CMT.

### HER-2

HER-2 is also considered an important tumor marker. HER-2 functions to regulate tumor growth, survival and differentiation. It is expressed in approximately 30% of CMT, which is the same percentage as in HBC [71–75].

In HBC patients, HER-2 overexpression is strongly related to decreased survival [76]. Approximately 30% of all HBC express HER-2 [77]. Therefore, an HER-2 targeted drug, trastuzumab, combined with chemotherapy is often used in the treatment of HBC.

In veterinary medicine, a positive correlation between HER-2 serum (determined by ELISA) and tissue (determined by IHC) expression was noticed [71]. There is also a positive correlation between HER-2 expression and tumor mitotic index, high histological grade and size [72, 74, 78]. It is considered to be a marker of poor prognosis [70, 73]. However, not all studies have confirmed this [66, 71, 72, 79], and no difference between HER-2 expression in benign and malignant tumors was seen [66, 71, 79]. Surprisingly, one study showed that dogs with malignant tumors expressing HER-2 showed a longer survival rate than HER-2-negative dogs [73]. Therefore, HER-2 may participate in tumor formation, but not necessarily in malignant transformation, or at least, it is not a good marker of malignancy.

As there is high homology between human and canine HER-2 antigens, antibody-based immunotherapy with trastuzumab or cetuximab seems to be promising in canine patients with HER-2 expression [75]. In regard to the contradictory research results in veterinary medicine, further studies should be performed [80].

## Hormone receptors

Estrogen and progesterone are the most studied biomarkers of CMT [10, 39, 81–101]. Estrogen and progesterone are essential for normal mammary tissue growth and development [97]. Both estrogen and progesterone are thought also to have an influence on tumor growth. The majority of CMT (benign and malignant) express ER and/or PR [86]. Additionally, prolactin and growth hormones were found to be involved in the process of tumor growth [94, 97].

HBC can either have a low or high steroid hormone expression. HBC with low expressions of ER and PR are not sensitive to endocrine therapy and are related to a poor prognosis, whereas HBC with a high expression of these steroid hormones have slightly better clinical outcomes [84, 92]. Both receptors are only detected in epithelial tumor cells [97]. Expression of ER and PR, together with HER-2, is evaluated to estimate HBC staging and improves prognostic accuracy. ER and PR targeted therapies, such as tamoxifen treatment, improve survival in women with hormone receptor-positive breast cancer [101].

In veterinary medicine, some studies have shown that the expression of one or both receptors was more frequent in benign tumors, and in general, its expression was related to a more favorable prognosis [87, 89, 91]. Though, in other studies, increased levels of estrogen, progesterone and prolactin were found in cases of malignant neoplasms [81, 95]. In one study, both serum and tissue steroid hormone expression (both determined by competitive EIA) was increased in patients with malignant tumors and a poor prognosis [95]. A recent study revealed that estrogen-negative (ER−) and progesterone-positive (PR+) tumors were correlated with a worse clinical outcome than ER+ and PR+ tumors, but at the same time, ER- and PR- tumors had the worst prognostic factor of all [86]. Another study showed that dogs with grade 2, ER− positive tumors as well as dogs with increased serum E2 level are likely to benefit from OHE [15].

Determination of both ER and PR expression should always be considered in cases of HBC as well as CMT. Nevertheless, sex hormone targeted therapy does not seem to play an important role in CMT, as it does in HBC, probably due to the appearance of multiple estrogenic side effects, such as vulva edema, purulent vaginal discharge and pyometra [102, 103]. However, in one study, safe doses of tamoxifen in canine mammary neoplasias were recommended [103]. Still, more studies concerning the efficacy of this selective estrogen-receptor modulator in the treatment of CMT must be carried out.

## Biomarkers of inflammation

Chronic inflammation is always present during neoplastic processes. Inflammatory cells secrete pro-inflammatory mediators, such as cytokines and chemokines, some of which promote angiogenesis and consequently tumor growth [104]. We only briefly describe the most commonly evaluated biomarkers of inflammation in CMT.

### Cox-2

The cyclooxygenase enzyme catalyzes the prostaglandin biosynthesis from arachnoid acid. Many studies have confirmed that prostaglandins play an important role in the tumor’s development. Deregulation of the enzymatic pathway of prostaglandin E2 formation is strongly related to neoplasm progression [105]. There are two isoforms: cyclooxygenase 1 (COX-1) and cyclooxygenase 2 (COX-2), but they have different biological functions. COX-1 is expressed in normal tissues and is responsible for the control of renal function, reproduction and, among other things, cytoprotection of the stomach. COX-2 is undetectable in normal tissues; it is expressed in tissue due to inflammatory reactions, growth factors, tumor promoters and oncogenes.

In human medicine, COX-2 overexpression is associated with the risk of tumor recurrence, advanced cancer stage, presence of metastases and poor overall survival in patients with HBC, as well as in ovarian, pancreatic, and gastric cancers, among others [106]. COX-2 inhibitors are used in the treatment of HBC [76].

In veterinary medicine, many studies have proven the presence of COX-2 expression in mammary tumors, as well as in some normal mammary tissues [5, 61, 104–115]. In mouse models of mammary neoplasias, COX-2 inhibitors suppress tumor growth [108]. The COX-2 expression (determined by IHC) is higher in cases of malignant CMT than in benign CMT [5, 105]. Tumors that express COX-2 may be treated with COX-2 inhibitors, such as meloxicam or piroxicam (they inhibit cell proliferation and angiogenesis) [111]. However, the mechanism of their antiproliferative effect is still unclear [111]. One study demonstrated that bitches with inflammatory mammary carcinoma treated with piroxicam had better outcomes than bitches treated with traditional chemotherapy [106].

In both human and veterinary medicine, selective COX-2 inhibitors are useful in the treatment of HBC, as well as CMT, but combination with other antitumor drugs is necessary. To obtain good treatment results with COX-2 inhibitors, the expression of COX-2 in tumors should be previously determined.

## Mutations of BRCA1 and BRCA2 genes

Among multiple gene mutations, the mutations of BRCA1 and BRCA2 turned out to play very important roles in the formation of both human and canine tumors of the mammary gland. The BRCA1 gene is located on human chromosome 17q21 and is expressed in many mammalian tissues. It encodes a nuclear phosphoprotein that participates in the regulation of the cell cycle of mammary epithelial cells. The loss of BRCA1 expression results in defective DNA repair, abnormal cell cycle, increased apoptosis and tumorigenesis. It was found that BRCA 1 is a tumor suppressor gene [116].

In human medicine, many gene mutations were found in cases of HBC. Mutations in BRCA1 and BRCA2 are hereditary, and affected women have a risk of 56–84% of developing HBC [116]. Statistically, approximately 5–10% of all breast cancers in women are due to mutations in BRCA1 and BRCA2 genes [117]. BRCA1 mutations are associated with tumors of a higher grade and may also indicate ovarian cancer. Interestingly, BRCA 2 was also found in cases of male breast cancer [117].

In veterinary medicine, hereditary patterns of CMT are still under investigation, but several breeds are thought to be predisposed to CMT [117]. In one study, mutations in ten different genes associated with breast cancer were evaluated in CMT (by iPLEX genotyping of sixty-three single nucleotide polymorphisms), but only BRCA1 and BRCA2 were significantly associated with the development of this tumor [117]. BRCA1 was especially associated with malignant tumors (reduced nuclear expression), which was also confirmed in previous studies [118]. There was no difference in BRCA2 expression between benign and malignant tumors [117]. However, in another study, reduced expression of BRCA2 was found in CMT compared to normal mammary tissues [116].

Although, BRCA1 and BRCA2 have been studied for a long time, little is still known about their exact mutations and the functional mechanism that reduces their expression in mammary neoplasias in both humans and dogs. Further studies of the canine genome and its mutations should be carried out.

## MiRNA

MiRNAs are small, non-coding molecules involved in the post-transcriptional negative regulation of gene expression. Numerous studies have indicated that the level of miRNA expression is altered in several types of cancer, even within the same tumor. However, different miRNA expression profiles can be observed for cancer’s various stages. MiRNAs were found to participate in nearly all important cellular processes, such as the regulation of cell proliferation, differentiation, angiogenesis, migration and apoptosis [119]. There is also strong evidence that miRNAs act as oncogenes or tumor suppressor genes [119, 120]. They can act as both serum or tissue biomarkers.

In human medicine, many studies have been done on miRNA expression in HBC. Several miRNAs were found to have diagnostic potential (miR-9, miR-10b and mir-17-5p), while others had a prognostic potential (mi-R-148a and miR-335) [121, 122]. Some miRNAs were found to have multiple roles in the diagnosis, prognosis and prediction of therapeutic response in HBC.

In veterinary medicine, a few analyses of the miRNA expression levels in tissues of canine mammary tumors (CMT) have been published [119, 120, 123, 124]. In one study, nine of ten miRNAs studied (miR-15a, miR-16, miR-17-5p, miR-21, miR-29b, miR-125b, miR-145, miR-155, miR-181b, let-7f) that are involved in human breast cancer appeared to have the same expression pattern in CMTs [119]. Different miRNA expression patterns (determined by qRT_PCR) have been observed in different tumor types: miR-15a and miR-16 showed a significant down-regulation in canine ductal carcinomas, while miR-181b, miR-21, miR-29b and miRlet-7f were strongly up-regulated in canine tubular papillary carcinomas [119]. In another study, the suitability of 16 miRNAs to distinguish between different stages of CMT was analyzed [123]. It was shown that metastatic cells differed from primary tumors cells in terms of mir-29b, miR-101, miR-125a, miR-143 and miR-145 levels of expression [123]. Recent studies have also indicated that microRNA expression differs significantly between metastatic and non-metastatic tumors, making it a good metastasis biomarker [120]. Studies of miRNA expression in canine mammary cancer cell lines have also shown its altered expression [124, 125].

Evaluation of miRNA is a very novel, but promising, method of cancer diagnosis. The fact that dogs follow similar miRNA expression patterns to those of humans suggests that further studies can be carried out on canine models. Recent studies in cancer patients suggest that multiple miRNA-based profiles may be very useful in the diagnosis and prognosis of both HBC and CMT, and miRNA-based drugs seem to be a promising targeted therapy for those tumors.

## Cancer stem cells

CSCs, also called tumor-initiating cells (TICs), are a small subpopulation of tumor cells that have the ability to renew themselves, as well as differentiate into different cancer cells [125]. They are thought to be responsible for drug, chemotherapy and radiotherapy resistance, tumor recurrence and metastasis [125–128]. The origin of CSCs is still unclear. It is thought that they result from the malignant transformation of normal stem cells, de-differentiation of mature cancer cells or induction of pluripotent cancer cells [126]. Identification and isolation of these cells has become an opportunity for new therapeutic strategies [127].

In human medicine, CSCs are well-described and identified by several molecular methods. They express several cell surface antigens, such as CD44, CD24 and EPCAM, which facilitate its identification. The CD44+/CD24− phenotype of CSCs has often been evaluated and was found to be related to a poor prognosis [128, 129].

It has been demonstrated that canine mammary CSC express similar surface markers to human breast CSC, and they are resistant to chemotherapy and radiotherapy [126]. The CD44+/CD24− phenotype of CSCs has been associated with high-grade canine mammary carcinoma [128]. In another study, a significant deregulation of 33 miRNAs was found in canine CSCs isolated from different CMC cell lines [127].

Due to the similar biological behavior of canine mammary and human breast CSCs, studies on canine models may result in useful breast cancer research. Nevertheless, the lack of a universal method for CSC detections, as well as the multiple phenotypes of CSCs and their heterogenic and dynamic nature, makes their exploitation as a diagnostic tool difficult to predict and assess.

## Circulating tumor cells

CTCs are tumor cells that are found in the peripheral blood, originating from either the primary tumor or its metastases. Therefore, they possess antigenic or genetic characteristics of specific tumor types [130]. Their presence is necessary for the development of distant metastases, so they may play an important role as biomarkers of a tumor’s metastatic potential.

In HBC, the detection of CTC is helpful in disease monitoring. CTCs can be counted in blood samples using CTC kits on the FDA-approved CellSearch System [131]. A correlation was found between the number of detected CTCs and a shorter overall survival in women with metastatic breast cancer [131, 132]. At the same time, decreasing the number of CTC was correlated with disease regression [133].

In veterinary medicine, just a few studies have been carried out so far, though a set of potential CTCs was found [130, 134]. The CTCs were identified through the expression of six specific genes: AGR2, ATP8B1, CRYAB, F3 IRX3 and SLC1A1 using DNA microarrays [134]. CRYAB was found to have the highest specificity and moderate sensitivity for CTC detection [130].

To understand the usefulness of these promising markers, further studies are required. However, CRYAB appears to be a good marker for detection of CTCs in dogs with metastasis of CMT.

## Conclusions

Over the past few decades, many studies concerning biomarkers of the mammary glands have been carried out in both human and veterinary patients. However, no ideal biomarker has yet been found. A perfect biomarker should be easily measured in the blood, and its concentrations should only be elevated in cases of malignant tumors. Second, it should be tumor-specific, and its measurement should give a clear prognosis. Serum biomarkers have the advantage over tissue biomarkers, as the procedure of measurement is non-invasive, and they show dynamic changes of physiological and pathological states before the clinical signs appear. Therefore, they may be used for the early detection of cancers.

Although many studies have been carried out concerning the clinical application of mammary tumor biomarkers, no productive conclusions have been drawn. Human biomarkers of HBC have proven to be detectable in CMT; therefore, this enables clinical diagnostic and treatment options for bitches suffering from this disease.

In both human and veterinary medicine, the most promising group of HBC and CMT biomarkers are miRNAs due to their high specificity and sensitivity. MiRNAs can act as oncogenes, as well as tumor suppressor genes, and different miRNA expression levels are altered in cases of both HBC and CMT. They can be measured in both blood and tissues, so they are very useful.

The evaluation of cancer stem cells as a tissue biomarker is also very novel, but due to their multiple phenotypes and lack of a universal method for their estimation, they are not a very precise biomarker. Nevertheless, in human medicine, this tissue biomarker helps to choose new therapeutic strategies, as many CSCs show resistance to radiotherapy as well as chemotherapy.

Circulating tumor cells may act as good serum biomarkers of tumor metastatic potential in humans, but in veterinary medicine, further studies must be done.

Recently, also collagen signatures turned out to be a good prognostic biomarker and potential target for treatment as its expression (collagen density, fiber width, length and straightness) inversely correlates with patient overall survival time [135].

The mutations of BRCA1 and BRCA2 in human medicine are hereditary and an indicator of a high possibility of high-grade breast cancer development. In dogs; however, similar mutations were found and seemed to be related with tumor formation have still not been proven to be hereditary. More detailed studies concerning gene mutation inheritance and the influence of BRCA1 and BRCA2 mutations on the grade of CMT should be done.

Up to now, it seems that the most studied and reliable biomarkers of CMT are Ki-67, EGFR, HER-2, ER, PR and COX-2, which can be detected in both serum and tissue samples using different molecular methods. In human medicine, those biomarkers are also evaluated; however, they are evaluated less frequently each time due to the application of newer biomarkers. Still, particularly if more than one of these biomarkers are evaluated, they can act as a reliable prognostic biomarker.

Notably, CEA and CA 15-3, which can be measured in tissues as well as in blood, while poorly studied, seem to be good biomarkers, especially for the early detection and prognosis of CMT. In human medicine, it has been proven that if those biomarkers are evaluated together, they have quite a high specificity and sensitivity, especially in the follow-up of the patients.

The evaluation of PCNA, protein p53, E-cadherin and VEGF, only if evaluated with the abovementioned biomarkers, may be useful. In summary, it is always recommended to evaluate more than one biomarker in order to obtain more reliable results.

Nevertheless, most of the studies were conducted on small groups of patients and/or very heterogeneous groups, using different molecular techniques. Therefore, the results are not truly reliable. More detailed studies should be carried out in the near future. So far, all abovementioned biomarkers seem to have certain prognostic potential in CMT. In cases of CMT, the evaluation of some biomarkers should always be considered.

## Abbreviations

BRCA1: breast cancer 1 gene
BRCA2: breast cancer 2 gene
CA15-3: cancer antigen 15-3
CEA: carcinoembryonic antigen
COX-1: cyclooxygenase 1
COX-2: cyclooxygenase 2
CMT: canine mammary tumors
CSCs: cancer stem cells
CTCs: circulating tumor cells
E-cadherin: epithelial cadherin
EGFR: endothelial growth factor receptor
ER: estrogen receptor
HER2: human epidermal growth factor receptor 2
HBC: human breast cancers
Ki-67: antigen Ki-67
miRNAs: microRNAs
OHE: ovariohysterectomy
p53: tumor protein p53
P-cadherin: placental cadherin
PCNA: proliferative cell nuclear antigen
PR: progesterone receptor
N-cadherin: neural cadherin
VEGF: vascular endothelial growth factor
